# Radiomics in preclinical imaging research: methods, challenges and opportunities

**DOI:** 10.1038/s44303-025-00104-z

**Published:** 2025-09-22

**Authors:** Vlora Riberdy, Alessandro Guida, James Rioux, Kimberly Brewer

**Affiliations:** 1 Department of Physics and Atmospheric Sciences, Dalhousie University, Halifax, NS Canada; 2 Biomedical MRI Research Laboratory (BMRL), IWK Health Centre, Halifax, NS Canada; 3 Biomedical Translational Imaging Centre (BIOTIC), NS Health, Halifax, NS Canada; 4 Department of Diagnostic Radiology, Dalhousie University, Halifax, NS Canada; 5 School of Biomedical Engineering, Dalhousie University, Halifax, NS Canada; 6 Department of Microbiology & Immunology, Dalhousie University, Halifax, NS Canada

**Keywords:** Imaging techniques, Image processing

## Abstract

Radiomics-based analyses are increasingly being applied to clinical studies. Radiomic features can be correlated with markers of disease severity or treatment success to improve early detection of disease and develop predictive models for therapeutic response. While radiomics has similar potential in preclinical research, its use in this context entails unique challenges. This paper provides an overview of the current state of radiomics in preclinical imaging, methodologies, challenges and future prospects.

## Introduction

Preclinical studies are an essential phase in the development of new drugs, therapies, and interventions. Preclinical imaging technologies play a vital role in advancing knowledge of diseases and testing new therapies in a non-invasive fashion. Methods such as computed tomography (CT), magnetic resonance imaging (MRI), ultrasound (US), positron emission tomography (PET), and single photon emission computed tomography (SPECT) can provide valuable information about disease progression and treatment efficacy. These methods enable longitudinal, non-invasive monitoring of disease treatment evaluations, and many of them permit cellular and molecular imaging to study underlying biological processes.

It is increasingly recognized that medical images are more than just pictures used for visual inspection and diagnosis. Images contain information about the underlying pathophysiology of the target tissue and can be converted into mineable high-dimensional data that can be correlated with outcomes to aid in diagnosis and prognosis^1^. This usually requires feature engineering: a mathematical transformation of raw data into specific features that are more strongly correlated to outcome variables, and which can therefore serve as actionable biomarkers.

While traditional feature engineering requires expertise in image processing and an understanding of the relevant biological and clinical processes, deep learning approaches are increasingly explored to identify relevant features directly from the underlying data^2^. These techniques have become popular for a variety of applications in medical imaging, including image reconstruction^3–6^, segmentation^7–9^, and diagnosis/classification^10–12^. Convolutional neural networks automatically learn patterns and extract features from images^13–15^, so there is no need for predefined features.

However, deep learning requires a substantial number of examples and therefore struggles in preclinical settings due to the intrinsic nature of the data, which is often characterized by high dimensionality and limited sample size. More conventional or “shallow” learning approaches must instead be used, but such models frequently encounter the so-called ‘curse of dimensionality’. These models may experience issues such as the bias-variance tradeoff, where improving the precision of the model on training data hampers its ability to perform on unseen data. To mitigate these problems, feature selection or dimensionality reduction before training remains crucial to retain the most informative input variables to avoid overfitting and improve model performance. Additionally, regularization techniques, such as L1 and L2 penalties, can help constrain model complexity and prevent overfitting.

Radiomics refers to the use of a particular standardized set of numerical engineered features that can be extracted from medical images, allowing a dataset containing several million voxels to be condensed into a few hundred radiomic features (RFs). These features, which range from simple statistical and shape measures to higher-order texture properties, can be computed from different imaging modalities across defined regions of interest (ROIs), such as tumors, metastatic lesions, and normal tissue^16^. Subsets of these features can then be used as inputs into various machine learning models.

Radiomics has been applied to various clinical studies, including tumor grading^17,18^, phenotyping^19–25^, tumor response to therapy^26–28^, making predictions on outcomes^29–32^ and identifying biomarkers for Alzheimer’s^33^. Radiomics has also been paired with genomics data to improve predictive outcomes^34^. Various reviews on the use of radiomics in clinical studies have been written for applications in glioblastoma^35,36^, Alzheimer’s^37^, liver metastasis^38^, rectal cancer^39^, and hepatic diseases^40^.

Radiomics applied to preclinical studies is a rapidly developing and evolving field. It has been used to aid in disease characterization and classification^41^, treatment response assessment^42^, drug development/optimization and discovery of biomarkers^43^. RFs can help identify phenotypic subtypes and explore tumor heterogeneity^44^. Longitudinal studies with animal models allow tracking of disease progression and treatment outcomes. Monitoring changes in RFs in a controlled animal model over time can help assess treatment efficacy or possibly identify early indicators of response. Some features may correlate with predictions of future disease characteristics or treatment outcomes. Preclinical studies allow for in-depth histologic investigations, and RFs may correlate with this data, further improving therapy development and our understanding of disease biology^45^. Radiomics could also be applied to preclinical studies to test novel therapies or drugs and identify early indicators of response or toxicity.

In general, while clinical and preclinical radiomics studies extract and analyze quantitative features, they differ in their context, goals and applications, depending on the area of research. While clinical studies often use radiomics to assist in diagnosis, prognosis and treatment planning, preclinical studies may focus on developing methodologies, studying mechanistic disease causes, and testing new imaging techniques or novel drugs and therapeutics. Preclinical radiomics studies are crucial for discovery research prior to implementing large-scale clinical studies by developing and validating methods in a controlled setting.

Compared to clinical studies, preclinical studies also have different advantages and disadvantages. While clinical studies often include hundreds or sometimes thousands of patients, this is generally not the case with preclinical studies, which complicates the detection of patterns with radiomics and other data-driven methods such as machine learning. Compared to clinical research, preclinical studies tend to include a wider range of biological information and can potentially employ multiple imaging modalities, introducing additional dimensionality and complexities to the data. Radiomics-based methods can assist in the evaluation of such data, but only if correctly applied and interpreted.

A PubMed search of “preclinical radiomics”, “animal radiomics” and “preclinical texture analysis” produced 48 papers published from 2015 to 2024. Table 1 summarizes some of these publications^46–55^, with the remainder summarized in the table in the Supplementary Material. In each case, a radiomics approach was used in various preclinical disease models using one or more imaging modalities. This review will discuss how preclinical radiomics is typically performed, how it was used in these studies, and how radiomics can best be incorporated into future studies to improve results.

**Table 1:** Summary of a subset of preclinical radiomics studies not mentioned in the text

	Imaging modality	Purpose	Number of animals	Timepoints
Becker^46^	MRI (T2w)	Correlation of texture features with liver tumor growth	10 (Mice)	5
Zinn^47^	MRI (T1w with contrast)	Causality between gene expression status and RFs	40 (Mice)	1
Bressem^48^	CT	Predict the presence of coagulation necrosis	8 (Pigs)	1
Gabrys^49^	CT	Determine if radiomic patterns in mice can be translated to human patients with interstitial lung disease	91 (Mice)	6
Spieler^50^	CT MRI	Predict lung inflammation associated with RT/IT	19 (Mice)	1
Zhang^51^	CT	Assess image quality on DL-based reconstruction compared to iterative reconstruction for dose reduction	1 (Sheep)	1
Rifi^52^	CT	Discover the biological meaning of RFs	Mice	1
Cheki^53^	CT	Find associations between RFs and proteins involved in metformin radiosensitivity	32 (Mice)	2
Kiser^54^	DCE MRI (T1)	Effects of anisotropic resolution on texture features	16 (Mice)	1
Zhang^55^	PET/CT	Differentiate T cell exhaustion status	24 (Mice)	2/5

The full table is included in the Supplementary Materials.

## Radiomics: General methods and considerations

### Radiomics pipeline

#### Image acquisition and preprocessing

**Fig. 1:**
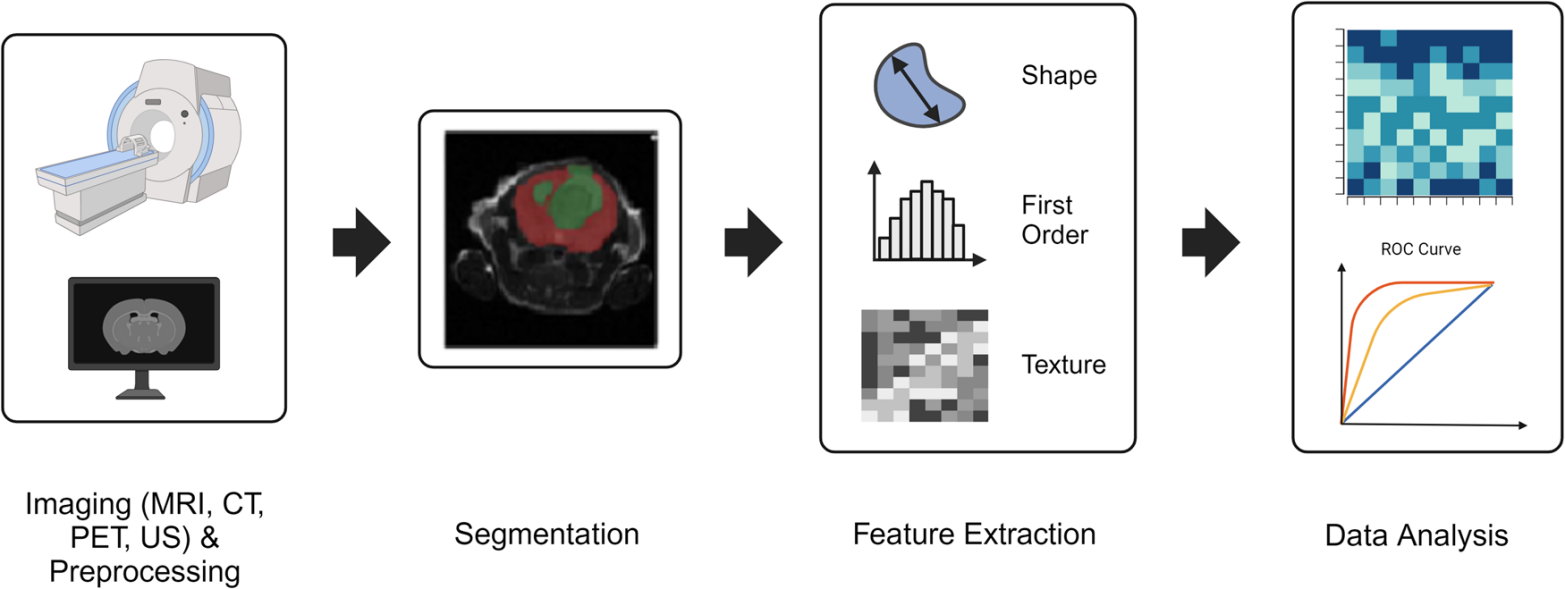
The radiomics process consists of several steps. The first is imaging and preprocessing. Radiomics can be applied to various imaging modalities. Preprocessing steps include artifact correction, normalizing image intensities, and noise reduction. The second step is segmentation of ROIs from which features are extracted. Segmentation can be manual, semi-automatic or automatic. Next, RFs are extracted from the ROIs. Typically, IBSI-compliant software is used, such as Pyradiomics. RFs are then used for data analysis. This can include correlations with biological parameters or input into machine learning algorithms. Created in BioRender. Brewer, K. (2025) https://BioRender.com/e51n133.

Figure 1 illustrates a typical preclinical radiomics pipeline involving several steps, the first of which is to acquire images. This pipeline is essentially identical to a clinical radiomics pipeline. Various imaging modalities were used in the preclinical radiomics studies in this review, and radiomics is easily applied to all these modalities. Some preprocessing of the image data may be required; the details of which will depend on the imaging modality. This can include artifact correction, image registration for multi-modal studies, normalizing image intensities to account for variations in acquisition parameters, and noise reduction. This preprocessing is often necessary to improve image quality, but it varies wildly between labs, even within a given modality. This pre-processing step may also vary between clinical and preclinical studies, particularly depending on the equipment manufacturer and/or software provider.

#### Image segmentation

The next step is the segmentation of ROIs, typically a tumor or pathology of interest, or other organs. Segmentation typically involves the creation of a binary mask whose value is one for regions inside the ROI and zero outside. It can be done manually, or with semi-automated or automated tools such as 3D Slicer (http://www.slicer.org), VivoQuant™ (Invicro, A Konica Minolta Company), ITK-SNAP (http://www.itksnap.org/pmwiki/pmwiki.php) and Matlab (MathWorks Inc.), to name a few. Some groups have developed deep learning-based segmentation algorithms to delineate ROIs for this purpose^43,56^.

#### Feature extraction

Segmentation is followed by the extraction of radiomic features from the ROIs using open-source or in-house developed software. Many labs have developed their own feature extraction pipelines, often using Matlab^57–59^. Unfortunately, many papers that use their own extraction code do not include detailed methodology, which can significantly hamper reproducibility. Pyradiomics, an open-source Python-based RF extraction software, is increasingly being used in the field^48,60,61^. Since Pyradiomics uses standardized mathematically defined features, it significantly improves experimental reproducibility. Most software packages typically allow users to configure various extraction parameters, such as preprocessing settings and binning methods, to tailor the extraction process to specific needs. These settings should be reported for reproducibility. Feature normalization (i.e., ensuring all features have a similar mean and standard deviation) may also be performed to provide a consistent scale, and it is often a requirement for several machine learning methods.

#### Analysis

Once the RFs have been extracted, the final step is a statistical analysis to explore the relationships between RFs and outcomes. This could be as simple as building correlation curves and looking for high levels of correlation between certain features and outcomes, or comparing whether there are significant differences in features between groups. These outcomes are defined by the researcher and will vary depending on the study’s goals. Another option is to use RFs as input into machine learning algorithms for classification or building predictive models. This is more complex and will be discussed in more detail later in this review.

### Radiomics features

“Core” RFs fall into three main categories: shape-based, first order and texture^1^. Size and shape-based features describe geometric properties of the ROI, such as elongation, sphericity, or surface area, and can be 2D or 3D^62^. First-order features come from the image gray-level histogram and describe distributions of intensity values within the ROIs^1^. These include features such as maximum, minimum, mean, and median. In medical imaging, texture refers to differences in gray-scale values within an ROI^63^. Texture features can be useful in representing the heterogeneity and spatial distribution of signal within an ROI. These include features derived from various gray-level matrices:

- The gray-level co-occurrence matrix (GLCM) describes spatial relationships of pixel/voxel pairs of a predefined gray-level intensity in different directions (pyradomics: radiomic features).
- The gray-level run length matrix (GLRLM) provides information about the spatial distribution of “runs” or continuous segments of pixels/voxels with the same gray level in multiple directions^62^.
- The gray-level size zone matrix (GLSZM) counts the number of groups (zones) of interconnected pixels/voxels with the same gray level^62^.
- A neighborhood gray-tone difference matrix (NGTDM) quantifies the sum of differences between a pixel/voxel’s gray level and its neighbors’ mean gray level within a predefined matrix^62^.
- The gray level dependence matrix (GLDM) quantifies the number of connected pixels/voxels within a certain distance that are dependent on the center voxel (pyradomics: radiomic features https://pyradiomics.readthedocs.io/en/latest/features.html).

Typically, the number of extracted “core” features across all types is on the order of 100–200. Some groups choose to apply various filters/transformations to their images before extracting RFs, which results in a larger number of features. For example, Panth et al.^44^ extracted features from unfiltered images, and after wavelet decomposition and Laplacian of Gaussian filtering, resulting in 625 RFs per image. Theek et al.^64^ applied wavelet filtering to their ultrasound images. In addition to their original unfiltered images, Eresen et al.^65^ extracted RFs from gradient-filtered and wavelet decomposition images. Starosolski et al.^66^ extracted logarithmically enhanced and wavelet features. Allphin et al.^67^ used wavelet decomposition and Brown et al.^68^ extracted RFs from wavelet-filtered cone-beam CT images.

### Dimensionality issues

Extracting RFs from filtered images provides more features to work with, but also contributes to the “curse of dimensionality”^69^. As the number of features or dimensions (*p*) increases, the sample size (*n*) needed to obtain reliable statistical estimates also increases^69^. The studies included in this review often had feature sets with hundreds of RFs, but much smaller sample sizes (*p* » *n*). Utilizing all features in a model with a small dataset can lead to overfitting, where the model performs well on the training data but fails to generalize to unseen data.

Some strategies can be used to mitigate this issue, which are applied between the feature extraction and data analysis steps. One example is feature selection, which identifies the most informative features to reduce the dimensionality of the dataset. Various techniques are available, such as filter methods, which include analysis of variance (ANOVA) and chi-squared, and wrapper methods, which include forward feature selection and recursive feature elimination^70,71^. Regularization techniques introduce a penalty term to the model’s objective function to promote more generalizable models^70^. Examples include ridge regularization and least absolute shrinkage and selection operator (LASSO) regularization^72^. Dimensionality reduction techniques help to reduce the number of input variables by transforming the original set of features into a new, lower-dimensional set. Increasing the sample size is another option, but it can be logistically challenging, especially for preclinical studies. Combining multiple sequential studies can help, but it takes time and requires compatible data.

### Feature standardization

Variability in the specific set of RFs used in a given study can hinder their widespread use and translation. As previously mentioned, early in the adoption of radiomics, many groups chose to develop their own features and tools for RF extraction, which limited reproducibility since these tools were not always properly benchmarked or distributed for wider evaluation. The Image Biomarker Standardization Initiative (IBSI) was formed to address these challenges: Zwanenberg et al.^73^ assessed RFs and standardized a set of 169 features, enabling verification and calibration of different software. Various IBSI-compliant software packages are now available for the extraction of standardized RFs. PyRadiomics^74^ is one of the most commonly used tools and was employed by several preclinical studies in this review. The medical image radiomics processor (MIRP) is another IBSI-compliant processor and was used by Muller et al.^75^. These software packages can also be used in clinical studies. Some groups still choose to develop their own software for RF extraction. Care must be taken when in-house software is used to ensure that feature values follow the same definitions as other radiomics software. IBSI guidelines should be followed to ensure reproducibility.

## Preclinical radiomics and machine learning

Machine learning is a branch of artificial intelligence that focuses on algorithms/models using statistical techniques to learn patterns from data, allowing them to make predictions or decisions based on unseen data. There are two main types—supervised and unsupervised learning. Supervised learning involves training with a labeled dataset to learn a mapping from input to output^72^. Common supervised learning models include linear regression, logistic regression, support vector machines (SVMs), and decision tree-based learners. Unsupervised learning involves finding patterns in unlabeled data^72^. Common unsupervised learning models include *K*-means clustering, hierarchical clustering, and principal component analysis. Both types of machine learning models can accept radiomic features as inputs. To ensure comprehensive reporting of prediction model studies, the Transparent Reporting of a multivariable prediction model for Individual Prognosis Or Diagnosis (TRIPOD) checklist^76^ was developed and should be followed. Radiomics studies can also be assessed with the radiomics quality score (RQS).

### Radiomics for classification

One widespread use of radiomics feature-based algorithms is classifying groups, e.g., distinguishing between benign and malignant tumors. As previously mentioned, Ni et al.^77^ compared six different machine learning classifiers, with RFs from 3D T_1_ MR images as input, to classify liver fibrosis stages in rat models. They found that SVM with feature regularization (LASSO) performed the best based on the area under the receiver operating characteristic curve and a fuzzy comprehensive evaluation method. Du et al.^78^ found that an SVM algorithm could discriminate between two groups of rat lung fetal maturity (terminal sac period and canalicular period) from US images of 67 fetuses from six rats. Muller et al.^75^ extracted MRI-derived RFs from two cohorts of head and neck squamous cell carcinoma tumor models and used them as input to a logistic regression model to predict tumor phenotype using leave-one-out cross-validation, which uses different portions of the data to train and test the model to avoid overfitting. The model was built with MR features alone, histology features, and a combination of both, with similar performances based on the area under the receiver operating characteristic (ROC) curves.

Eresen et al.^79^ extracted RFs from T_1_ and T_2_ MR images of rabbit livers after irreversible electroporation. Three Random Forest classification models were developed using T_1_, T_2_, and T_1_ + T_2_ features to differentiate between irreversibly and reversibly electroporated zones. This was done with region-based (zones) and pixelwise classification. Combined T_1_ + T_2_ did not significantly improve performance for the region-based classification but did for the pixelwise classification. Additionally, three Random Forest regression models were developed using T_1_, T_2_ and T_1_ + T_2_ features to evaluate the correlation between RFs and histological tumor biomarkers. Ruiz-Espana et al.^80^ performed animal and human studies to assess whether functional MRI texture features could be used to differentiate alcohol use disorder from healthy controls. Six models were evaluated, and the best performance was seen with a random forest model.

### Predicting treatment outcomes

Machine learning algorithms can also be used to predict outcomes, such as responses to treatment. Bao et al.^60^ found that a random forest classifier performed best at detecting early protein changes after incomplete thermal ablation using RFs extracted from CEUS images. Mihaylov et al.^81^ utilized RFs from CT and MRI combined with complete blood count data to predict the abscopal effect in 15 mice implanted with Lewis lung carcinoma cells and treated with a combination of RT and the PD-1 inhibitor immunotherapy. CT and MR features were used to build a binary logistic regression model, separately and combined with the complete blood count features. Mice were split into two groups, one for model generation and one for validation. The validation set revealed an area under the receiver operating characteristic curve above 0.84 for each model. Roy et al.^82^ studied a patient-derived xenograft mouse model of triple-negative breast cancer and treatment with docetaxel. RFs were extracted from PET/CT images obtained before treatment and four days after the start of treatment. Three machine learning models with baseline RFs (prediction) and changes in RFs between 4 days post-treatment and baseline (therapy assessment) were tested. The naïve Bayes model performed best in both scenarios compared to classification, regression tree, and SVM.

## Example preclinical radiomic applications

### Feature dependence on imaging parameters

One common application of radiomic features in preclinical studies involves observing how RFs respond to changes in other imaging variables, and whether these differences are statistically significant. Alsyed et al.^58^ observed how radiomic texture features extracted from FDG-PET images vary with contour size and acquisition time using the determinant of correlation matrices, indicating that imaging itself can change RF data. Roy et al.^83^ studied the sensitivity of RFs extracted from MR images to noise, image resolution and tumor volume and found certain features were sensitive to these changes. Dutta et al.^56^ developed an automated image segmentation algorithm and studied the effects of tumor boundaries on RFs. RFs extracted from automatically segmented regions showed better reproducibility than those extracted from manually segmented regions. Benfante et al.^61^ used one-way (ANOVA) and post hoc Tukey Honest Significant Difference (HSD) tests to determine if there were significant differences in RFs between acquisition times of a new ^64^Cu-labeled PET tracer as a way of evaluating its biodistribution in different organs. They found that several features varied significantly in the bladder and liver over different acquisition times. These results indicate that RFs can depend on imaging variables, such as segmentation, image resolution and acquisition time, which should be considered when designing studies or when combining data from multiple studies.

### Uncommon imaging techniques

**Fig. 2:**
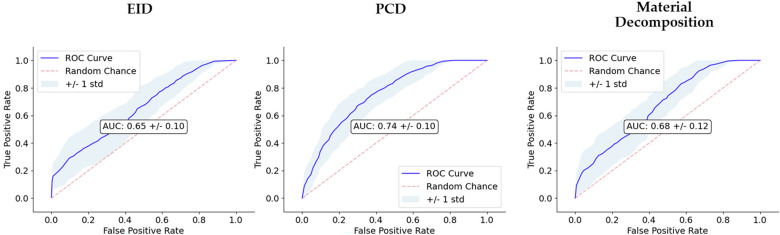
Comparison of classifier performance via receiver operator characteristic curves for EID, PCD and material maps from Allphin et al.^67^. Area under the curve (AUC) is reported in the center of the plots. The dashed lines represent a no-skill classifier. PCD provided the best performance out of the three RF sets. This figure was originally published in Allphin et al.^67^ and reproduced with permission via the Creative Commons Attribution 4.0 International License (https://creativecommons.org/licenses/by/4.0/).

Many preclinical imaging techniques can be used to study and characterize diseases, even some that are not used in clinical contexts. Allphin et al.^67^ utilized an iodine nanoparticle (NP)-enhanced CT using an energy integrating detector (EID) and spectral micro-CT using a photon counting detector (PCD). Features were extracted from EID-CT, PCD-CT and spectral decomposition maps of iodine, Photoelectric effect, and Compton Scattering. RFs were able to discriminate between high and low lymphocyte burden of sarcomas with a logistic regression classification model. Figure 2 shows the receiver operator characteristic curves for each modality from this paper.

### Radiomics and contrast agents

Contrast agents are used in various imaging modalities to enhance the visibility of structures or processes. Gadolinium is a commonly used contrast agent for MRI, with paramagnetic properties that alter relaxation times in nearby tissues. After IV administration, it accumulates in tissues or structures with increased vascular permeability, such as tumors or inflammatory lesions^84,85^. Holbrook et al.^43^ implanted soft tissue sarcomas in mice and extracted RFs from T_1_-weighted images pre and post-Gd-DTPA injection, and T_2_-weighted images before and after radiation treatment. The differences in RFs extracted from the tumor and peritumoural area pre- and post-radiation were found to predict tumor recurrence by utilizing a simple neural network and SVM.

**Fig. 3:**
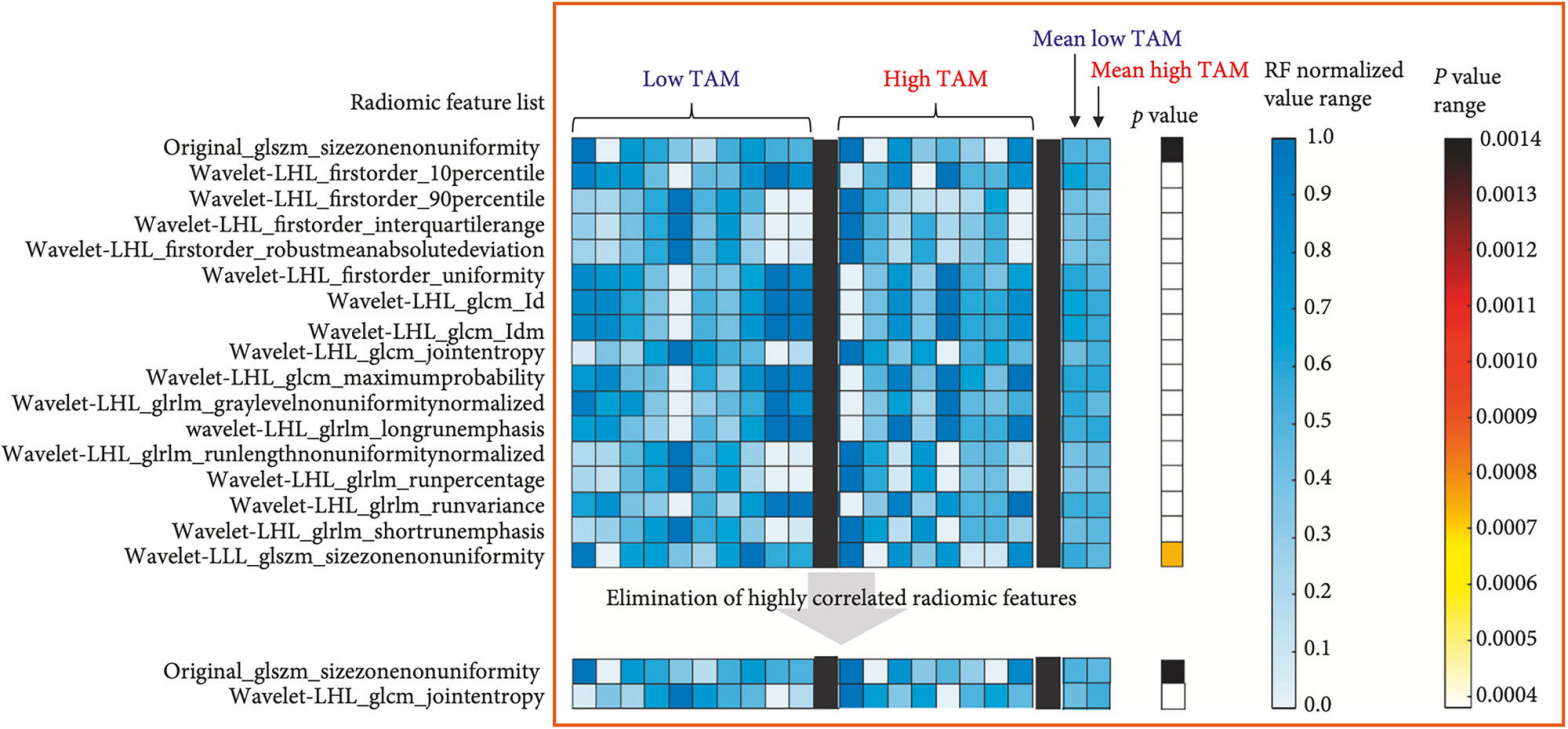
Radiomic analysis on nanoparticle contrast-enhanced CT from Starosolski et al.^66^. A total of 17 RFs were found to be able to differentiate low and high tumor-associated macrophage (TAM) burden. Highly correlated features were removed, and 2 remained, which were then used as input in a linear classifier. Each column shows a vector of normalized RF values representing individual tumor samples. Mean values of each RF were calculated for the low and high TAM groups. The Kruskal–Wallis test was used to identify RFs that differentiated low and high TAM groups; *p* < 0.05 was considered statistically significant. This figure was originally published in Starosolski et al.^66^ and reproduced with permission via the Creative Commons Attribution License.

As previously mentioned, NPs are another type of contrast agent for medical imaging. They tend to accumulate in tumors because of enhanced permeability and retention^86,87^ and are also taken up by phagocytic cells such as macrophages and monocytes^88–90^. Devkota et al.^91^ utilized NP-enhanced delayed CT, CT angiography (CTA) and T_2_ MRI to study cellular immunotherapy directed against myeloid-derived suppressor cells (MDSCs). Some texture features could differentiate between control groups and groups treated with natural killer cell immunotherapy, which was determined through a Kruksal–Wallis test with Bonferroni corrections for multiple intergroup comparisons. Starosolski et al.^65^ extracted RFs from NP-CT images to differentiate between low and high tumor-associated macrophage (TAM) burden in a transgenic mouse model of neuroblastoma. Conventional image-derived metrics, such as tumor volume and CT attenuation, could not differentiate between burdens, but two RFs were identified to enable differentiation between high and low TAMs utilizing a supervised machine learning module. Figure 3 shows the radiomics analysis steps they took to identify these features. The first feature to enable differentiation was GLSZM—size zone non-uniformity (from an unfiltered image). The RF quantifies the size of areas of connected voxels that share the same gray level intensity, and size zone non-uniformity describes the variability of zone sizes across the image (pyradomics: radiomic features). The second feature was GLCM—joint entropy from the wavelet low-high-low (LHL) filtered image. The wavelet transform represents the image in different frequency components, and GLCM joint entropy measures the amount of randomness in intensity values (pyradomics:Radiomic features). Both of these texture features can be linked to the distribution of TAMS throughout the brain, although further biological testing would be required to describe the exact relationship.

### Radiomics beyond cancer

Although most commonly applied preclinically in cancer, radiomics can be applied to animal studies of other diseases. Ni et al.^77^ extracted RFs from MR images of healthy controls and rat models of liver fibrosis. Six binary classification tasks were performed to differentiate between ranges of fibrosis stages. Various machine learning models were tested with two feature reduction techniques. Singh et al.^92^ used radiomic texture features for subtyping four genetically distinct rat models of autism spectrum disorder via hierarchical clustering. Multicompartment diffusion-weighted MRI (MC-DWI) was performed and fit to a neurite orientation dispersion and density index (NODDI) model. RFs were extracted from the three NODDI signals (ODI, NDI, CSF) from the amygdala, hippocampus and corpus striatum. They found that RFs from the ODI signal of the left and right hippocampus improved discrimination of subtypes compared to the mean signal intensity. Zhang et al.^93^ studied RFs extracted from US images of a rat model of muscle atrophy to differentiate between the model and healthy controls. They compared SVMs with a multiple-layer perceptron classifier. Aweda et al.^94^ compared the biodistribution of a SARS-COV-2 monoclonal antibody, sotrovimab, and a modified equivalent using ^89^Zr-based PET/CT radiomics to evaluate the effect of the modification in healthy cynomolgus monkeys via a linear mixed effect model.

### Radiomics in ultrasound

Although not as commonly seen as CT, MRI and PET, radiomics can also be applied to US imaging. Various clinical studies have extracted RFs from ultrasound images for input into machine learning algorithms^95–97^. Contrast-enhanced US (CEUS) utilizes microbubbles injected into the bloodstream, which resonate at higher frequencies and produce strong echoes, resulting in enhanced contrast. Theek et al.^64^ extracted RFs from CEUS B-mode images to differentiate between three xenograft mouse tumor models using a linear support vector machine. Bao et al.^60^ found that arterial-phase CEUS RFs can identify protein changes immediately after incomplete tumor ablation in mice using a random forest binary classifier.

### Radiomics changes with radiotherapy

**Fig. 4:**
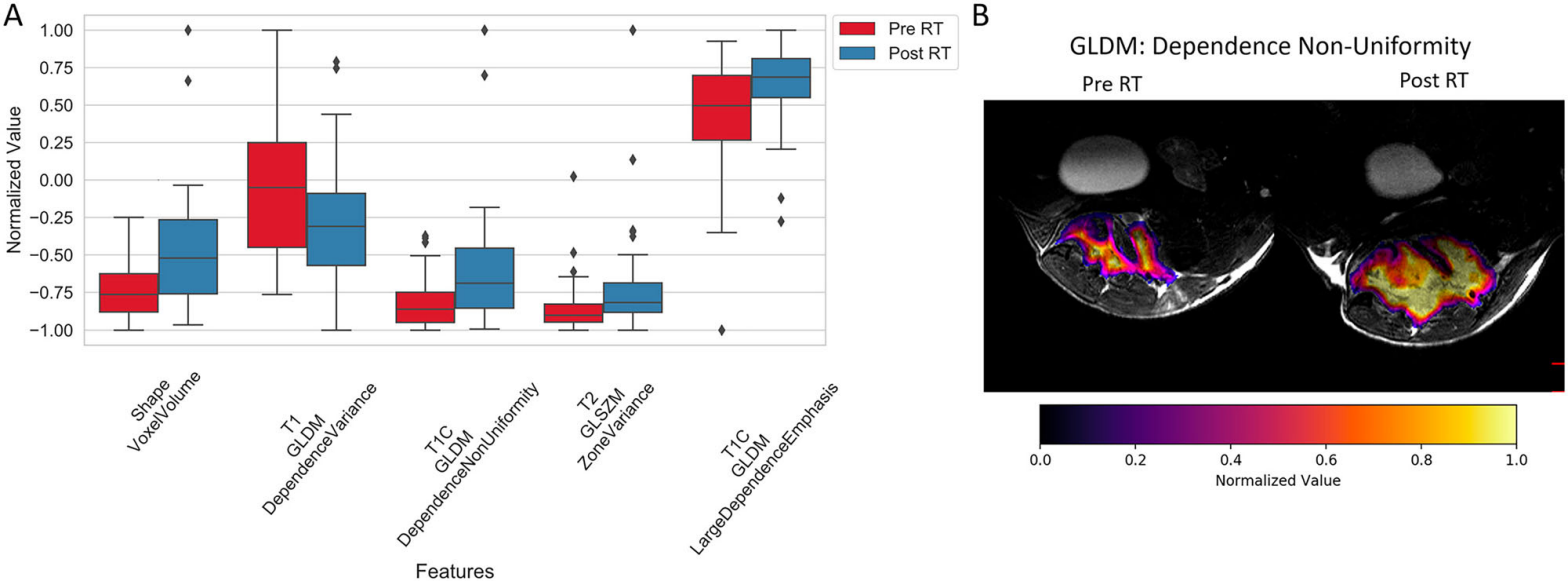
Changes in RFs extracted from MR images after RT from Holbrook et al.^43^. **A** Statistically significant differences before and after RT were found in 5 RFs. **B** Spatial maps of one RF before and after RT (Gray level dependence matrix–dependence non-uniformity). This figure was originally published in Holbrook et al.^43^ and reproduced with permission via the CC BY-NC-ND license (http://creativecommons.org/licenses/by-nc-nd/4.0/).

Radiation therapy (RT) is a standard treatment for different types of cancers, and the physical changes that occur in the tissue as a result can also cause changes in RFs. Panth et al.^44^ found significant changes in several CT-derived RFs four days post-irradiation via a Wilcoxon rank sum test. Along with using neural networks to predict tumor recurrence, Holbrook et al.^43^ performed MR imaging one day before and one week after a single dose (20 Gy) of RT and found that multiple RFs changed significantly using paired *t*-tests. Figure 4 shows the significant changes in five RFs and spatial maps of the changes, in which texture-based RFs are computed on a per-voxel basis to present a more detailed visualization of texture variation within the ROI.

### Correlations with Histopathology

RFs have also been investigated for correlations with histopathology findings. Ahmad et al.^45^ extracted features from pre-RT and immunotherapy (IT) CT and T1-weighted MR images of five mice implanted with breast cancer cells (4T1). Tumors were harvested, and tissue was stained for Ki-67, a cellular proliferation marker. Pearson correlation measures found that seven CT features and 24 MR features were highly correlated with the Ki-67 score. Eresen et al.^66^ studied a mouse model of pancreatic ductal adenocarcinoma and found that MRI texture features were correlated with fibrosis percentage and immunostains CK19 and Ki-67 between control mice and mice treated with a dendritic cell vaccine. Correlations with histological biomarkers indicate the non-invasive prognostic potential of RFs.

## Study design and analysis considerations for preclinical research

Preclinical studies often have far fewer subjects than clinical studies. While some larger preclinical cohorts have been reported (Muller et al.^75^ utilized a total of 114 mice over 3.5 years), this is not logistically feasible for many laboratories. Preclinical animal studies can be resource-intensive, requiring specialized facilities, personnel, and animal care costs. Ethical guidelines and regulations also aim to minimize the number of animals used. Larger animals are more expensive and difficult to house, requiring more specialized facilities than murine models. With fewer subjects, the statistical power of such studies is limited, making results difficult to generalize. The choice of statistical test is critical and should be compatible with the study sample size.

When utilizing machine learning methods with a small dataset, there are risks of overfitting, leading to reduced performance and generalizability. Furthermore, the high dimensionality of radiomics features paired with small sample sizes in preclinical studies can lead to overfitting and poor generalizability. The number of features extracted can range from hundreds to thousands, depending on feature categories and applied image filters. Machine learning models must be selected appropriately. Simple models such as logistic regression, linear regression or decision trees may be best for small sample sizes^72^. Effective feature selection or dimensionality reduction techniques are essential to dimensionality problems. Small data sets make it difficult to set aside data to test the model’s ability to generalize to unseen data. This is important to ensure the evaluation reflects how the model would behave in a real-world setting and to get an unbiased estimate of the model’s performance ability. As an alternative, cross-validation techniques, such as *k*-fold and leave-one-out, can often be used^98^.

It is also important to distinguish between development and evaluation phases when working with limited data. Some studies use only a two-way split (train/test), rather than a more appropriate three-way split into train, validation, and test sets (e.g., refs. ^43,60^). This can lead to the test set being used multiple times to guide model selection, introducing bias and overfitting. Ideally, the test set should be held out and used only once at the very end of the modeling process to obtain an unbiased estimate of performance. Additionally, while less common in preclinical work, some studies do report validating their models on truly external or out-of-distribution datasets (e.g., ^81^^,^^59^). This kind of evaluation provides important insight into the model’s generalizability, particularly when the external data comes from different sources.

As previously mentioned, deep learning can also be used either in conjunction with or as an alternative to radiomics. However, deep learning models typically benefit from larger and more diverse datasets to make the model generalizable to unseen data. Because preclinical datasets tend to be smaller, deep learning tends not to be the best choice for analysis, unless it can be applied across large numbers of animals. The difficulty in applying deep learning methods to preclinical studies also scales with the complexity of the data, particularly with multiple timepoints and modalities.

In longitudinal designs with multiple imaging timepoints, RFs can be extracted from images at each timepoint to observe temporal changes caused by a specific treatment or biological process. Panth et al.^44^ extracted RFs from CT images at three timepoints to compare differences between groups before and after RT treatment. Collie et al.^99^ used RFs to study responses to lung irradiation using sheep (exposed and contralateral lungs). CT imaging was performed at three timepoints before and after radiation. They found a significant time-dependent influence on GLCM homogeneity. Benfante et al.^61^ extracted RFs from PET images acquired 1, 4, and 24 h after administration of a chelator to characterize its biodistribution. With multiple imaging timepoints, temporal changes in RFs can be captured to better understand treatments and disease progression. There are various statistical techniques for analyzing longitudinal data, such as mixed-effect regression, generalized estimating equation models, and multivariate ANOVA^100,101^. When using machine learning algorithms to classify or make predictions, longitudinal data can be tested separately to maintain independent observations. Machine learning methods are available for longitudinal data as well^102^.

Multimodal or multiparametric imaging can capture different aspects of tissue properties or physiological processes. RFs can be extracted from all images to determine which gives the best results. Ahmad et al.^45^ extracted RFs from CT and T_1_-weighted MR imaging, and RFs from both were found to be correlated with histopathological features. Mihaylov et al.^81^ compared RFs extracted from CT and T_1_-weighted MRI as input into machine learning algorithms combined with blood count measures to determine which performed better in predicting the abscopal effect in a tumor model. The MR combined with blood count features performed the best. Lee et al.^103^ performed radiomics on CT and US images and used them as input in machine learning algorithms to differentiate between stages of sialadenitis. Both models had excellent performance metrics. However, the increase in RFs available for analysis from multiple imaging types can bring about challenges in selecting an optimal subset, especially in model training for machine learning (the curse of dimensionality). Data from each modality can be tested separately or in combination. Feature reduction techniques should be used to avoid overfitting and model complexity.

## Discussion and recommendations

Preclinical radiomics involves the application of in-depth data analysis to preclinical imaging studies. Features are extracted and analyzed to uncover meaningful patterns and correlations that can provide insight into diagnosis, treatment and understanding of disease. There are several directions preclinical radiomics can take. RFs can be used for early detection and classification of disease type^41^, allowing for early intervention. New therapies are being developed and tested, for which radiomics can provide quantitative measurements of treatment responses^66,104^. Integrating radiomics data with histopathology data can offer a more comprehensive view of disease processes and provide insight into molecular mechanisms of diseases^75^.

Radiomics is a valuable tool for preclinical imaging analysis with many advantages. By extracting a large number of features from images, we can get a comprehensive view of tissue characteristics. The pairing of radiomics and machine learning has become popular in clinical studies and could be applied preclinically as well. RFs can be used as input variables to train models to predict outcomes and to differentiate between disease types. Since radiomics can be applied to various imaging modalities and contrasts, multi-modal studies can provide a more comprehensive view of preclinical models from which RFs can be extracted. These developments have the potential to enhance our ability to study diseases and develop effective treatments in preclinical settings.

Radiomics provides a non-invasive way of monitoring different processes, such as disease progression and treatment, particularly when used with molecular imaging technologies investigating biological processes. Since RFs can be correlated with histological, flow cytometry or other biological assay results, they could provide a non-invasive alternative to tissue characterization. By pairing radiomics with machine learning algorithms, there is the potential to aid in classification and prediction studies. Radiomics can be applied to many different imaging modalities and disease models.

However, the use of radiomics also has disadvantages that can introduce difficulties to these studies, which must be addressed. Table 2 details several recommendations for improving the implementation of radiomics in preclinical imaging. The small sample sizes used in preclinical imaging due to ethical or logistical reasons can often pose an issue, especially in combination with the high-dimensional nature of radiomics data. Various feature reduction techniques are needed to remove correlated and redundant features and decrease the risk of overfitting. Cross-validation and resampling methods can also help. Dimensionality is further increased with multi-modality imaging as well as longitudinal data. Care must be taken when using multi-modal and longitudinal data, and the data should be assessed to see if it should be combined or tested separately to avoid introducing bias and affecting the validity of the statistical analysis with data dependence.

**Table 2:** Issues and recommendations for improving the implementation of radiomics in preclinical imaging studies

Issue	Recommendations
Small sample size	• Feature reduction • Cross-validation • Resampling methods
Multi-modality	Test separately and combined
Longitudinal data	Test timepoints separately to avoid bias Longitudinal-based models or machine learning
Regions of interest	Use semi-automatic or automatic segmentation tools Choose based on the disease model
Reproducibility	Use an IBSI-compliant software (Pyradiomics, MIRP, IBEX)

The low spatial resolution of certain modalities, such as PET, compared to the size of the lesions or organs being studied in small animal models, can pose limitations. Partial volume effects and blurring can compromise the accuracy of some RFs. In many cases, groups utilize specialized animal scanners that allow for higher resolution. Since RFs can change with ROI size and imaging parameters, these should be optimized and consistent within a study. Clinical studies have shown that images taken on different scanners can cause variability in the RFs^105–107^, and the same can also be true for preclinical studies, so the scanner type and imaging protocols should be consistent as well. This may be easier with preclinical studies, as they tend to be performed at a single facility, but compiling data across facilities is more difficult.

Because manual segmentation of regions can introduce inter-observer variability, automatic and semi-automatic methods are recommended in clinical studies^108–111^. These methods benefit preclinical radiomics studies as well. This is a burgeoning area of study with many labs using machine learning and transfer learning to develop accurate segmentation tools^112^. The ROIs should be chosen carefully and can depend on the disease model being studied. For example, in a cancer treatment study, the tumor may not be the only important region to focus on. If the tumor may metastasize, then these distant regions should be examined, as well as relevant areas that may be linked to immune responses (i.e. spleen and lymph nodes) Many diseases are systemic and restricting analysis to a small region limits the information available, Unfortunately, multiple ROIs can also exacerbate the multi-dimensional nature of radiomics studies, as is the case for multi-modality and longitudinal studies. Regions can be tested separately to determine the best results to minimize the issue. The same problems can arise in clinical radiomics studies as well.

Finally, applying radiomics to biological studies requires explanation to help validate them, but it can be difficult to interpret many RFs biologically. Further studies into the biological meaning of RFs are needed. Preclinical studies may be the ideal way to do this due to the control over experimental conditions and specific protocols. This can be helpful when looking at how RFs change due to treatments, disease progression or changes in image parameters. Studies need to be conducted on translating preclinical radiomics studies to clinical studies—are the features transferable between animal models of disease and humans? Gabrys et al.^49^ studied the transferability of radiomic signatures from micro-CT images of preclinical mouse models of interstitial lung disease to human interstitial lung disease. Multivariate models to predict disease were trained on preclinical data and tested on patient data. They found good model performance in the patient validation sets and re-optimized patient models. More studies like this are needed to determine the extent to which the transferability of RFs between animal models and human studies is dependent on imaging modalities, models or case contexts. Certain factors, such as imaging modality, disease model, and image acquisition parameters (i.e. spatial resolution and reconstruction algorithms) may influence RF robustness and generalizability. These studies are essential if radiomics is to be considered a translational tool for biomarker discovery.

With regard to reproducibility, the IBSI has given a standardized set of RFs to extract from images, and various IBSI-compliant software is available. This makes radiomics more accessible to groups with less expertise in programming for image processing. Platforms like GitHub (http://github.com) make it easier for groups to share their data and code, which can help others develop their radiomics studies, and should be encouraged whenever possible. Some groups are also creating more standardized tools to manage preclinical data, such as PIXI [pixi.org]. Developing radiomics approaches that could be integrated into these platforms would enable more rapid proliferation and sharing of results.

## Conclusion

Medical imaging technologies have become essential tools in clinical and preclinical research. Radiomics has added to the value that medical imaging can provide by enabling the extraction of more complex information from medical images than is immediately visible. Preclinical radiomics studies have assessed the reliability of radiomic features and used them to create classification and predictive models. Addressing ongoing challenges related to standardization and reproducibility is crucial to improving the utility of radiomics in preclinical studies. With ongoing advancements in medical imaging techniques, preclinical radiomics can contribute significantly to understanding diseases and their treatment.

## Supplementary information

Supplementary Material

## Data Availability

No datasets were generated or analyzed during the current study.
