# Supplementary material for: Radiomics in preclinical imaging research: methods, challenges and opportunities
Source: Npj Imaging. 2025 Sep 22;3:45. doi: 10.1038/s44303-025-00104-z (PMC12454655; doi:10.1038/s44303-025-00104-z)
Supplement: Supplementary file 1 — Supplementary Material [file 44303_2025_104_MOESM1_ESM.docx]

**Radiomics in Preclinical Imaging Research: Methods, Challenges and Opportunities**

Vlora Riberdy^1,2^, Alessandro Guida^3,4^, James Rioux^1,3,4^, Kimberly Brewer^1,2,3,4,5,6 *^

^1^Department of Physics and Atmospheric Sciences, Dalhousie University, Halifax, Nova Scotia, Canada

^2^Biomedical MRI Research Laboratory (BMRL), IWK Health Centre, Halifax, Nova Scotia, Canada

^3^Biomedical Translational Imaging Centre (BIOTIC), NS Health, Halifax, Nova Scotia, Canada

^4^Department of Diagnostic Radiology, Dalhousie University, Halifax, Nova Scotia, Canada

^5^School of Biomedical Engineering, Dalhousie University, Halifax, Nova Scotia, Canada

^6^Department of Microbiology & Immunology, Dalhousie University, Halifax, Nova Scotia, Canada

Corresponding Author Email: [brewerk@dal.ca](mailto:brewerk@dal.ca)

Manuscript Category: Review

Supplementary Table 1: Summary of preclinical radiomics papers.

| Timeline | 3 imaging timepoints (pre, 4 days post RT, TV = 500 mm^3^) | 5 imaging timepoints (before injection of cells, 4, 8, 12, 16 and 20 days after | 1 imaging timepoint, each mouse images 4 2 times at 2 positions | 1 imaging timepoint | Images were re-binned into 4 PET acquisition times (50-55 min, 55-60 min, 60-65 min, 65-70 min) |
| --- | --- | --- | --- | --- | --- |
| Software | In-house | In-house (Matlab) | Unknown | Unknown | In-house (Matlab) |
| Number of Animals | 33  (Mice) | 10  (Mice) | 14  (Mice) | 40  (Mice) | 8  (Mice) |
| Purpose | Genetic changes with phenotypic consequences influence features  Change in radiomic features due to RT | Correlation of texture features with liver tumor growth | Differentiate 3 tumor xenograft mouse models | Causality between gene expression status and RFs | Effect of segmentation volume and acquisition time on radiomic features |
| Imaging Modality | CT | MRI (T2w) | CEUS | MRI (T1w with contrast) | PET/CT |
| Disease Model | HCT116 doxycycline inducible GADD34 | MC-38 colon cancer cells | Tumor xenograft models (A413, MLS, A549) | PDX – patient derived glioma stem cells | Mammary carcinoma xenografts (4T1) |
|  | Panth 2015 | Becker 2018 | Theek 2018 | Zinn 2018 | Alsyed 2019 |

| Timeline | 1 imaging timepoint | 1 imaging timepoint after microwave ablation | 1 imaging timepoint | Imaging at 1 and 3 weeks after start of DC vaccination treatment | 1 imaging timepoint 1 week after RT |
| --- | --- | --- | --- | --- | --- |
| Software | In-house | Pyradiomics | PyRadiomics | Unknown | PyRadiomics |
| Number of Animals | 5  (Mice) | 8  (Pigs) | 20  (Mice) | 16  (Mice) | 42  (Mice) |
| Purpose | Discriminate between responding and non-responding tumors (radioimmunotherapy) | Predict the presence of coagulation necrosis | Differentiating treatment group from untreated group | Texture features for detection of early IT response and overall survival | CNN segmentation  Features indicative of RT  Prediction of primary tumor recurrence |
| Imaging Modality | CT & MRI (T1) | CT | NP contrast- enhanced CT  MRI (T2) | MRI (T2) | MRI (T1 w/ Gd, T2) |
| Disease Model | TNBC (4T1 cells) | Microwave Ablation | MDSC TME | PDAC (KPC mice) | Soft tissue sarcoma |
|  | Ahmad 2020 | Bressem (2020) | Devkota  (2020) | Eresen (2020) | Holbrook (2020) |

| Timeline | 1 imaging timepoint per mouse after TMZ treatment | 2 imaging timepoints – baseline and 2 weeks after treatment | 1 imaging timepoint | 1 imaging timepoint | 5 imaging timepoints |
| --- | --- | --- | --- | --- | --- |
| Software | In-house (Matlab) | PyRadiomics | Matlab | PyRadiomics | Matlab |
| Number of Animals | 63  (Mice) | 13-14/group/PDX  (Mice) | 6+  (Mice) | 80  (Mice) | 4  (Shetland Sheep) |
| Purpose | Distinguishing between treated and control mice | Combine protein profiling and radiomics to identify tumors responsive to ABT-199 | Assess sensitivity of RFs to noise, resolution and tumor volume | Detect early protein changes after incomplete thermal ablation | Determine RF differences between timepoints after radiation in exposed and contralateral lungs |
| Imaging Modality | MRI (T2w) MRSI | FDG PET/CT | MRI  (T1, T2) | CEUS | CT |
| Disease Model | GL261 | PDX CRC0076 and CRC0344 | PDX – 6 TNBC subtypes | HCT-26 colorectal adenoma tumor cells | Radiation-induced lung inflammation |
|  | Nunez (2020) | O’Farrell (2020) | Roy (2020) | Bao (2021) | Collie (2021) |

| Timeline | 1 imaging timepoint | Imaging at 1 and 3 weeks after start of DC vaccination treatment | 3 consecutive days of imaging starting 1 day after radioimmunotherapy | 1 imaging timepoint | 1 imaging timepoint, 4 days after NP injection |
| --- | --- | --- | --- | --- | --- |
| Software | In-house (Matlab) | Unknown | In-house | IBEX | PyRadiomics |
| Number of Animals | 49  (Mice) | 16  (Mice) | 19  (Mice) | 97  (Rats) | 18  (Mice) |
| Purpose | Develop automated segmentation pipeline and assess sensitivity of RFs to tumor boundaries | Detection of DC vaccine treatment effects and outcomes | RFs to predict the abscopal effect | Identify optimal feature selection and classifier to classify liver fibrosis stage | Differentiate tumors based on TAM burden |
| Imaging Modality | MRI (T1w, T2w) | MRI (T1w & T2w) | CT  MRI | MRI (T1w) | NP contrast- enhanced CT |
| Disease Model | PBX – TNBC (6 subtypes) | PDAC | Lewis Lung Carcinoma | Liver fibrosis | Neuroblastoma |
|  | Dutta (2021) | Eresen (2021) | Mihaylov (2021) | Ni (2021) | Starosolski (2021) |

| Timeline | 1 imaging timepoint, 5 days after NP injection | Acquisition 1h, 2h, 3h, after chelator administration | 1 imaging timepoint | 1 imaging timepoint | 6 imaging time points (different mice) |
| --- | --- | --- | --- | --- | --- |
| Software | PyRadiomics | PyRadiomics | Matlab | Unknown | Z-Rad |
| Number of Animals | 25  (Mice) | 9  (Mice) | 67  (Rats) | 14  (Rabbits) | 91  (Mice) |
| Purpose | Differentiate tumors based on lymphocyte burden | Testing biodistribution of ^64^Cu-labeled chelator in different organs | Develop a classification model to evaluate fetal lung maturity | Differentiate between ablation zones and correlate with histological tumor markers | Determine if radiomic patterns in mice can be translated to human patients with interstitial lung disease |
| Imaging Modality | Micro-CT  Spectral micro-CT  (NP enhanced) | Micro-PET/CT | US | MRI (T1w & T2w) | CT |
| Disease Model | Soft Tissue Sarcoma | - | Rat fetal lungs | VX2 liver tumors | Lung Fibrosis |
|  | Allphin (2022) | Benfante (2022) | Du (2022) | Eresen (2022) | Gabrys (2022) |

| Timeline | 1 imaging timepoint after irradiation | 2 imaging timepoints – baseline and 4 days following start of therapy | 1 imaging timepoint | Imaging was done post-mortem | 1 imaging timepoint before RT+IT |
| --- | --- | --- | --- | --- | --- |
| Software | MIRP | In-house | Pyradiomics | Pyradiomics | In-house |
| Number of Animals | 114  (Mice) | 29  (Mice) | 21  (Mice) | 11  (Rats) | 19  (Mice) |
| Purpose | Discrimination between cell types using MRI and histological features | Identify RFs to predict response to therapy (docetaxel) in TNBC and implement in a co-clinical trial | Differentiate between two breast cancer derived xenograft models | Autism subtyping | Predict lung inflammation associated with RT/IT |
| Imaging Modality | MRI (T1) | PET/CT | US | MRI (DWI) | CT  MRI |
| Disease Model | Radioresistant and radiosensitive HNSCCs | PDX – TNBC | PDX – breast cancer | ASD | Lewis Lung Carcinoma |
|  | Muller (2022) | Roy (2022) | Sanchez (2022) | Singh (2022) | Spieler (2022) |

| Timeline | 1 imaging timepoint | 4 imaging timepoints | 1 imaging timepoint | 1 imaging timepoint | 1 imaging timepoint (retrospective study) | 2 imaging timepoints (before and after treatment) |
| --- | --- | --- | --- | --- | --- | --- |
| Software | Python-based package | Matlab toolkit | Pyradiomics | Unknown | Pyradiomics | Pyradiomics |
| Number of Animals | 1  (Sheep) | 49  (Rats) | Unknown  (Mice) | 6  (Monkeys) | 9  (Mice) | 32  (Mice) |
| Purpose | Assess image quality on DL-based reconstruction compared to iterative reconstruction for dose reduction | Evaluation of muscle atrophy | Discover the biological meaning of RFs | Evaluate impact of LS modification on VIR-7831 monoclonal antibody | Assess reproducibility and repeatability of RFs | Find associations between RFs and proteins involved in metformin radiosensitivity |
| Imaging Modality | CT | US | CT | ^89^Zr-based PET/CT | Cone beam-CT | CT |
| Disease Model | - | Hindlimb unloading model | CT26, 4T1 EMT6 | - | NSCLC (A549, H460) | Breast Cancer |
|  | Zhang (2022) | Zhang (2022) | Rifi 2022 | Aweda (2023) | Brown (2023) | Cheki (2023) |

| Timeline | 1 imaging timepoint | 2 imaging timepoints, before and after surgery | 2 imaging timepoints (before and after access to alcohol) | 7 imaging timepoints (2 experiments) | Single post-injury study: 0, 2, 4, 7, 10 or 14 days after injury  Longitudinal: 2, 4, 7, 10 and 14 days post-injury |
| --- | --- | --- | --- | --- | --- |
| Software | Pyradiomics | LIFEx | Matlab | IBEX | Pyradiomics |
| Number of Animals | 16  (Mice) | 16  (Rats) | 36  (Rats) | 24  (Mice) | 23  (Rats) |
| Purpose | Effects of anisotropic resolution on texture features | Differentiate between stages of sialadenitis | Identify biomarkers to identify alcohol use disorder brain network alterations | Differentiate T cell exhaustion status | Prediction of recovery process |
| Imaging Modality | DCE MRI (T1) | CT  US | fMRI | PET/CT | CT |
| Disease Model | Glioma - Gl261 cells | Sialadenitis | Alcohol use disorder | Lewis lung carcinoma | Skeletal muscle injury |
|  | Kiser (2023) | Lee (2023) | Ruiz-Espana (2023) | Zhang (2023) | Eleftheriadis (2023) |
| Timeline | 1 imaging timepoint | 1 imaging timepoint | 2 imaging timepoints | Up to 6 imaging timepoints | 4 imaging timepoints |
| Software | Pyradiomics | Pyrariomics | Z-rad software | Radiomics Frontier | Pyradiomics |
| Number of Animals | 16 (mice) | 26 (mice) | 30 (mice) | 24 (mice) | 24 (mice) |
| Purpose | Investigate effects of inter-observer and inter-software delineation variabilities of radiomics outputs | Compare radiomics output from two preclinical CT-based scanners | Assess whether changes in radiomics signatures can classify the antifibrotic response to nintedanib | Develop a radiomic signature to predict early treatment response | Differentiate between tumour implanted hemisphere to non-tumour implanted hemisphere and apply features to a clinical dataset |
| Imaging Modality | CBCT | CBCT  MicroCT | microCT | MRI (T2) | CT |
| Disease Model | NA | NA | Lung fibrosis | Patient-derived organoid endometrial cancer | Glioblastoma |
|  | Brown (2023) | Brown (2024) | Lauer (2024) | Espedal (2024) | Connor (2024) |
| Timeline | 2 imaging timeppoints | 4 imaging timepoints |  |  |  |
| Software | Pyradiomics | Pyradiomics |  |  |  |
| Number of Animals | 16 (mice) | 72 (mice) |  |  |  |
| Purpose | Evaluate the biodistribution of a novel Ga-68 labeled radiopharmaceutical | Differentiate radiation pneumonitis (early stage) and pulmonary fibrosis (late stage) |  |  |  |
| Imaging Modality | PET/CT | CBCT |  |  |  |
| Disease Model | Human epidermoid carcinoma A431 cell line | Radiation-induced lung injuries |  |  |  |
|  | Pavone (2024) | Brown (2024) |  |  |  |
